# Postoperative improvement in patient‐reported outcomes after neutral alignment medial open wedge high tibial osteotomy for medial compartment knee osteoarthritis in patients aged ≥70 years versus younger patients

**DOI:** 10.1002/jeo2.12035

**Published:** 2024-05-21

**Authors:** Mitsuharu Nakashima, Tsuneari Takahashi, Tomohiro Matsumura, Katsushi Takeshita

**Affiliations:** ^1^ Department of Orthopedic Surgery Haga Red Cross Hospital Moka Japan; ^2^ Department of Orthopedic Surgery Ishibashi General Hospital Shimotsuke Japan; ^3^ Department of Emergency and Critical Care Medicine Jichi Medical University Shimotsuke Japan; ^4^ Department of Orthopaedic Surgery, School of Medicine Jichi Medical University Shimotsuke Japan

**Keywords:** age‐related outcomes, knee osteoarthritis, neutral alignment medial opening‐wedge high tibial osteotomy

## Abstract

**Purpose:**

To compare the postoperative clinical and radiological outcomes in patients aged ≥70 years following neutral alignment medial opening‐wedge high tibial osteotomy (NA‐MOWHTO) for medial compartment knee osteoarthritis (KOA) to those observed in younger patients.

**Methods:**

The data of patients who underwent NA‐MOWHTO for medial compartment KOA between September 2018 and June 2022 were retrospectively analysed. The patients were categorised into groups Y (<70 years) and O (≥70 years). Age, sex, Kellgren–Lawrence classification, pre‐ and postoperative mechanical axis, weight‐bearing line ratio, medial proximal tibial angle, preoperative Tegner Activity Score and pre‐ and postoperative Lysholm scores were compared between the groups.

**Results:**

Overall, 81 patients (60 and 21 in groups Y and O, respectively) who underwent NA‐MOWHTO were included in this study. No significant differences were found in patient characteristics between the two groups, except for the preoperative Tegner Activity Score, which was significantly higher in group Y than in group O (3 [2–4] vs. 2 [2–2], respectively; *p* = 0.011). The two groups exhibited no significant differences in pre‐ and postoperative knee alignments. Postoperatively, Lysholm scores improved significantly in both groups without significant differences. Additionally, no correlation was found between age and pre‐ and postoperative Lysholm scores.

**Conclusions:**

The postoperative improvement following NA‐MOWHTO for medial compartment KOA is comparable in patients aged ≥70 and younger.

**Level of Evidence:**

Level III, Retrospective comparative study.

**Abbreviations**
HTO: high tibial osteotomy
IRB: Institutional Review Board
KOA: knee osteoarthritis
MA: mechanical axis
mPTA: medial proximal tibial angle
NA‐MOWHTO: neutral alignment medial opening‐wedge high tibial osteotomy
WBL: weight‐bearing line

## BACKGROUND

High tibial osteotomy (HTO) is a widely used surgical treatment for medial compartment knee osteoarthritis (KOA) with varus deformities in relatively young and active patients [9]. Considering the recent improvements in surgical techniques and internal fixation materials, this technique is used even for older patients with promising outcomes [6, 11, 14, 15]. However, Fitoussi et al. reported that the revised risk factor for patients with medial compartment KOA aged <70 years who underwent valgus HTO was the oldest age group (60–69 years), likely due to osteoporosis [4]. Additionally, no consensus exists on the optimal alignment for medial opening‐wedge HTO (MOWHTO). Although the mechanical axis (MA) has traditionally targeted valgus alignment for some time [5, 7], concerns exist regarding the associated complications [3]. Atkinson et al. reported that correction to near‐neutral alignment rather than excessive valgus alignment is sufficient [1]. Many older people desire to work and enjoy sports; therefore, we expect an increase in the demand for neutral alignment MOWHTO (NA‐MOWHTO) in patients aged ≥70 years. We hypothesised that postoperative improvement in patients aged ≥70 years who underwent NA‐MOWHTO for medial compartment KOA was comparable to that of younger patients. Therefore, we conducted a retrospective study to compare the postoperative clinical and radiological outcomes in patients aged ≥70 years who underwent NA‐MOWHTO for medial compartment KOA with those in younger patients.

## METHODS

### Patient selection

The study was approved by the Institutional Review Board of the author's institution, and the requirement for informed consent from individual participants was waived. All patients included in this retrospective comparative study received standard treatment. The following were the inclusion criteria: (1) patients with medial compartment KOA with Kellgren–Lawrence classification grade 2–4, (2) those who underwent NA‐MOWHTO between September 2018 and June 2022 and (3) those who could be followed up for 1 year after NA‐MOWHTO. In contrast, patients who could not be followed up for 1 year after NA‐MOWHTO were excluded from this study. Patients were categorised based on their age into younger (Y; <70 years) and older (O; ≥70 years) age groups.

### NA‐MOWHTO procedure

**Figure 1:**
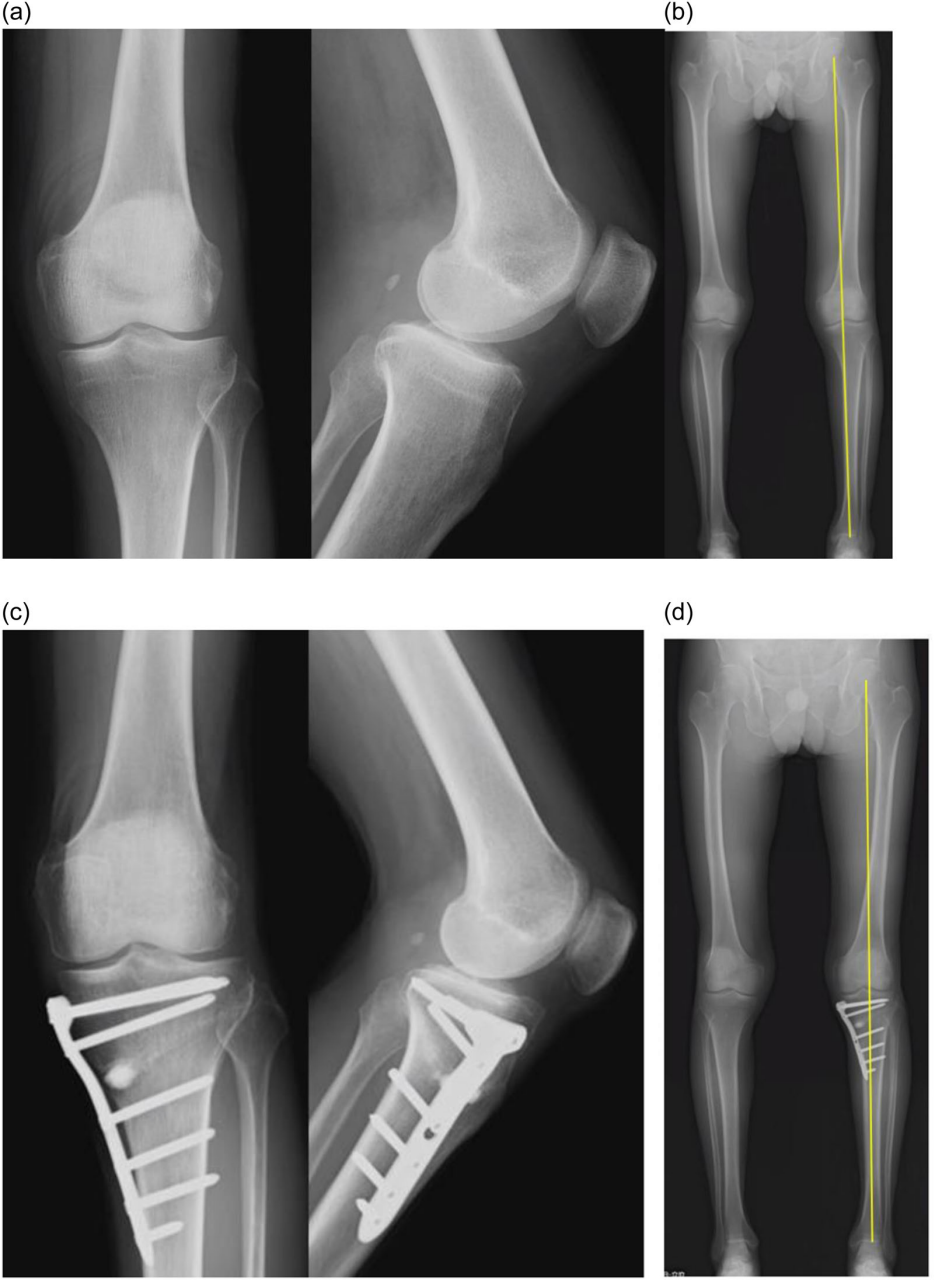
Radiological findings. (a) Preoperative radiograph. (b) Preoperative full‐length radiograph of the lower extremities with the Mikulicz line; mechanical axis (MA): 42%. (c) Radiograph 1 year postoperatively. (d) Postoperative 1‐year full‐length X‐ray of the lower extremities with the Mikulicz line: MA: 56%.

Preoperative anteroposterior standing long‐leg radiographs were used for preoperative planning. The postoperative weight‐bearing line (WBL) was targeted at a point that was 55% lateral to the transverse diameter of the tibial plateau. Next, the width of the medial horizontal osteotomy was determined accordingly. NA‐MOWHTO was performed as previously described [17]. Medial horizontal osteotomy was gradually opened to the desired correction angle [16], and the medial tibia was fixed with the TomoFix® anatomical medial high tibial plate (DepuySynthes). A spreader was positioned closer to the posterior cortex to minimise tibial slope alteration. The intraoperative MA of the lower limb was set at 55% [10] and assessed using a long alignment rod [18]. Figure 1 shows the pre‐ and postoperative radiographs. The patient was unloaded until 2 weeks postoperatively and was allowed only ange of motion training, 1/2 load from 2 weeks and full load at 4 weeks.

### Clinical and radiological evaluations

Age, sex, Kellgren–Lawrence classification, pre‐ and postoperative MA, WBL ratio, medial proximal tibial angle (mPTA), preoperative Tegner Activity Score and pre‐ and postoperative Lysholm scores [12] were evaluated. All radiographic measurements were taken by two observers (the first and the corresponding authors). Inter‐rater reliability for X‐ray measurements was good, with an intraclass correlation coefficient of >0.80. We compared the Lysholm score, preoperative Lysholm score, Lysholm score at 1 year postoperatively and ΔLysholm score (the difference between pre‐ and postoperative scores at 1 year) between groups Y and O, with ΔLysholm score as the primary outcome.

### Statistical analyses

Continuous data are presented as mean and standard deviation, and comparisons were made using Student's *t* test. Categorical data were compared using *χ* ^2^ and Fisher's exact tests. Ordinal data are presented as median and range, and comparisons were made using the Mann–Whitney *U* test. Student's *t* test was used to evaluate the differences in the pre‐ and postoperative Lysholm scores, MA and WBL ratios and mPTA between the groups. The *χ* ^2^ and Fisher's exact tests were employed to evaluate the differences in sex and the Kellgren–Lawrence classification between the groups. Furthermore, the Mann–Whitney *U* test was used to evaluate the differences in the preoperative Tegner Activity Scores between the groups.

All statistical analyses were performed using EZR software [8]. A priori sample size calculation for the primary outcome was performed, with statistical significance set at *p* < 0.05. Among the 81 patients who underwent NA‐MOWHTO, 60 and 21 were assigned to groups Y and O, respectively. The minimum sample size for an *α* error of ≤0.05, *β* error of ≤0.20, and Cohen's effect size of 0.80 [2] was calculated using G*Power 3.1 (Franz Paul) to be 54. A post hoc power was calculated using an *α* error of 0.05 and 81 patients in the two groups, and the actual power was 86.1%. Furthermore, we explored the relationship between age and pre‐ and postoperative alignments as well as Lysholm scores using Pearson's correlation coefficient.

## RESULTS

**Figure 2:**
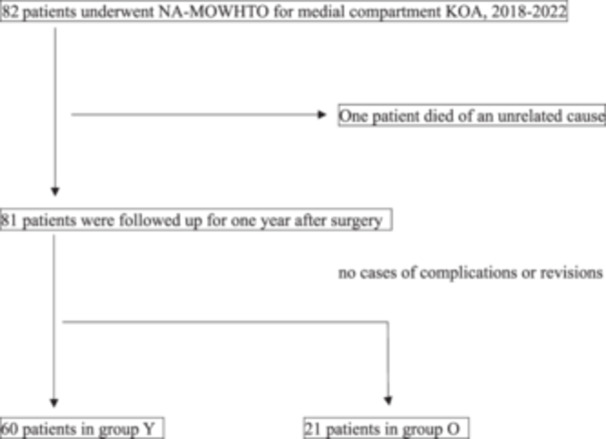
Study flow chart. Group Y: Younger group, age <70 years. Group O: Older group, age ≥70 years. KOA, knee osteoarthritis; NA‐MOWHTO, neutral alignment medial opening‐wedge high tibial osteotomy.

In total, 82 patients underwent MOWHTO for medial compartment KOA between September 2018 and June 2022. Specifically, all patients underwent NA‐MOWHTO. One patient died of an unrelated cause within 1 year postoperatively, and 81 who were followed up for 1 year postoperatively were retrospectively analysed using an electronic medical database. No cases of complications or revisions occurred. Finally, the 81 patients were categorised into the Y (<70 years) and O (≥70 years) groups, with 60 and 21 patients, respectively (Figure 2).

Table 1 summarises the patient characteristics. The mean age of the patients was 58.8 ± 7.2 and 72.2 ± 2.9 years in groups Y and O, respectively. Sex (male/female) was 24/36 and 5/16 in groups Y and O, respectively (*p* = 0.29). No significant differences were found in patient characteristics between the two groups, except for the preoperative Tegner Activity Scores, which were significantly higher in group Y (3 [2–4]) than in group O (2 [2–2]), (*p* = 0.011).

**Table 1:** Patient characteristics.

	Group Y (N = 60)	Group O (N = 21)	*p* Value
Age (years)	58.8 ± 7.2	72.2 ± 2.9	
Sex (male/female)	24/36	5/16	0.29
Kellgren–Lawrence classification			0.37
Grade 2	29	13	
Grade 3	24	5	
Grade 4	7	3	
Preoperative Tegner Activity Score (level)	3 [2–4]	2 [2–2]	0.011

Table 2 compares the pre‐ and postoperative alignments and Lysholm scores between the two groups. No significant differences were found in the radiological outcomes between the two age groups. Although no significant differences were found in the Lysholm scores between the two groups, the scores indicated a significant improvement postoperatively.

**Table 2:** Comparison of pre‐ and postoperative alignment and Lysholm scores.

	Group Y (*N* = 60)	Group O (*N* = 21)	*p* Value
Preoperative MA and WBL ratio (%)	29.4 ± 9.2	27.4 ± 11.0	0.42
Preoperative mPTA (°)	85.1 ± 2.4	86.0 ± 2.4	0.14
Preoperative Lysholm score	58.7 ± 16.7	58.0 ± 12.9	0.87
Postoperative MA and WBL ratio (%)	54.2 ± 7.6	51.0 ± 11.1	0.14
Postoperative mPTA (°)	89.5 ± 2.6	90.3 ± 2.6	0.21
Postoperative Lysholm score	89.0 ± 8.5	87.7 ± 11.9	0.58
ΔLysholm score	30.4 ± 17.6	29.7 ± 16.0	0.88

Abbreviations: MA and WBL ratios, mechanical axis, and weight‐bearing line ratios; mPTA, medial proximal tibial angle; Postoperative Lysholm score, Lysholm score at 1 year postoperatively; Δ Lysholm score, the difference between preoperative and postoperative 1 year.

Furthermore, no correlation was found between age and pre‐ and postoperative Lysholm scores (Table 3).

**Table 3:** Relationship between age and pre and postoperative Lysholm scores.

	*r*	CI	*p* Value
Preoperative Lysholm score	−0.093	−0.31 to 0.13	0.41
Postoperative Lysholm score	−0.091	−0.30 to 0.13	0.42
Δ Lysholm score	0.036	−0.18 to 0.25	0.75

Abbreviation: CI, confidence interval.

## DISCUSSION

The most important finding in this study was the confirmation of comparable postoperative improvement in patients aged ≥70 years versus younger patients following NA‐MOWHTO for the medial compartment KOA.

Previous studies on MOWHTO with rigid long plates have reported that age did not affect the clinical and radiological outcomes [6, 11, 14, 15]. Goshima et al. compared the postoperative clinical and radiological outcomes after MOWHTO and concluded that the age of >65 years did not influence outcomes. They retrospectively studied 60 knees after MOWHTO; 26 knees of patients aged >65 (68.7 ± 2.9) years were compared with 34 knees of those aged <65 (56.2 ± 7.5) years. Similar to our surgical technique, a TomoFix® plate was used; however, it differed in that no bone graft or substitute artificial bone was placed, and the WBL was aimed at a point 65%–70% lateral on the transverse diameter of the tibial plateau. Their results at the final follow‐up were consistent with ours as the patient‐reported outcome measures (JOA and Oxford knee scores) and postoperative alignment (femorotibial angle, WBL ratio, tibial slope and Blackburne–Peel ratio) did not differ between the two groups [6]. Saito et al. stated that MOWHTO, using a stable plate fixation system combined with a bone substitute, is a reliable procedure that provides excellent outcomes in patients aged ≥70 years. They retrospectively studied 78 knees after MOWHTO; 39 knees of patients aged ≥70 years were compared with 39 knees of those aged <70 years. A TomoFix® plate and bone substitute composed of hydroxyapatite was used, similar to our surgical technique; however, it also differed because the WBL was aimed to achieve 10° of anatomical valgus. At the final follow‐up, no significant differences were found in the Knee Society Scores and standing femorotibial angle between the two groups [15], which aligns with our present findings.

In contrast, Allison et al. reported that among patients aged <70 years who underwent valgus HTO for medial compartment KOA, the age of 60–69 years (oldest age group) was a revised risk factor, with osteoporosis being the likely cause. They retrospectively studied 43,537 patients aged <70 years who underwent HTO for medial compartment KOA from 2011 to 2020 to identify revised risk factors. Obesity, rheumatoid arthritis, joint chondrocalcinosis and the age of >60 years were identified as the revised risk factors. Furthermore, they considered that osteotomy consolidation might be more challenging in individuals aged 60–69 years due to osteopenia and osteoporosis, potentially resulting in reduced tolerance for conservative treatment [4].

No consensus exists on the optimal alignment in MOWHTO. The MA has been aimed at a point 62% lateral to the transverse diameter of the tibial plateau to decrease the medial tibiofemoral contact pressure in traditional OWHTO [5]. However, several complications may be associated with valgus alignment after OWHTO. Felson et al. stated that valgus malalignment increases the risk of radiographic progression, KOA incidence and lateral cartilage damage [3]. Recent studies have different opinions from those that emphasise valgus alignment, which is defined as >3° of valgus medial femoral‐tibial angle. Martay et al. established that finite element models might successfully simulate various HTO realignments. Correcting the WBL to 55% tibial width (1.7–1.9° valgus) optimally distributes medial and lateral stresses/pressures [13]. Furthermore, Atkinson et al. reported that HTO with an approximately neutral correction improves the medial compartment articular cartilage composition without compromising the lateral and patellar compartments [1]. Therefore, this study was performed in NA‐MOWHTO.

This study has some limitations. First, it was a retrospective study and not a randomised trial. Therefore, a potential bias may exist in the results of our patient cohort.

Second, all MOWHTO surgeries were exclusively performed using NA‐MOWHTO by a single consultant knee surgeon. Therefore, the absence of a control group makes it unclear whether NA‐MOWHTO yields better outcomes than valgus‐aligned MOWHTO. Only short‐term results have been reported, and long‐term follow‐up is required. Finally, since no postoperative computed tomography scan was performed, there was insufficient confirmation of hinge fractures, which is a concern in older patients.

## CONCLUSIONS

Postoperative improvement in patients aged ≥70 years following NA‐MOWHTO for medial compartment KOA is comparable to that in younger patients.

## AUTHOR CONTRIBUTIONS

**Mitsuharu Nakashima**: Study conception and design; data acquisition; data analysis and interpretation; article drafting; critical revision of the article for important intellectual content. **Tsuneari Takahashi**: Study conception and design; data acquisition; data analysis and interpretation; article drafting; critical revision of the article for important intellectual content. **Tomohiro Matsumura**: Article drafting; critical revision of the article for important intellectual content. **Katsushi Takeshita**: Article drafting; critical revision of the article for important intellectual content. All authors have contributed significantly to the study, approved the article and agreed with the submission. All authors read and approved the final manuscript.

## CONFLICT OF INTEREST STATEMENT

The authors declare no conflict of interest.

## ETHICS STATEMENT

This study was performed in accordance with the Declaration of Helsinki and was approved by the Jichi Medical University Bioethics Committee for Medical Research (Approval ID:22‐166). This was a retrospective study. All patients received standard treatment, and the requirement for informed consent from individual participants was waived.

## Data Availability

Data and materials for this study are available from the corresponding author upon reasonable request.
